# Interferon Induced Focal Segmental Glomerulosclerosis

**DOI:** 10.1155/2016/6967378

**Published:** 2016-10-26

**Authors:** Yusuf Kayar, Nuket Bayram Kayar, Nadir Alpay, Jamshid Hamdard, Iskender Ekinci, Sebnem Emegil, Rabia Bag Soydas, Birol Baysal

**Affiliations:** ^1^Department of Internal Medicine, Bezmialem Vakif University, Istanbul, Turkey; ^2^Bagcilar Education and Research Hospital, Department of Family Medicine, Istanbul, Turkey; ^3^Department of Internal Medicine, Division of Nephrology, Istanbul University, Istanbul, Turkey

## Abstract

Behçet's disease is an inflammatory disease of unknown etiology which involves recurring oral and genital aphthous ulcers and ocular lesions as well as articular, vascular, and nervous system involvement. Focal segmental glomerulosclerosis (FSGS) is usually seen in viral infections, immune deficiency syndrome, sickle cell anemia, and hyperfiltration and secondary to interferon therapy. Here, we present a case of FSGS identified with kidney biopsy in a patient who had been diagnosed with Behçet's disease and received interferon-alpha treatment for uveitis and presented with acute renal failure and nephrotic syndrome associated with interferon.

## 1. Introduction

Behçet's disease is an inflammatory disease of unknown etiology which involves recurring oral and genital aphthous ulcers and ocular lesions as well as articular, vascular, and nervous system involvement [1]. Interferon is used in ocular involvement of Behçet's disease. Renal injury from interferon is typically mild and includes proteinuria, mild azotemia, and abnormalities in urinary findings [2]. There are few case reports of development of FSGS due to interferon therapy in patients with Behçet's disease [3]. Here, we present a case of focal segmental glomerulosclerosis (FSGS) in a patient who had been diagnosed with Behçet's disease and received interferon-alpha treatment for uveitis and presented with acute renal failure and nephrotic syndrome associated with interferon.

## 2. Case Report

**Figure 1:**
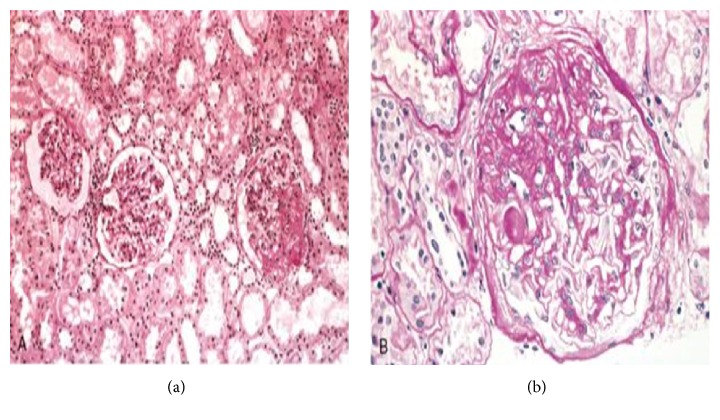
(a) A glomerulus with collapsing FSGS exhibits global wrinkling and retraction of the glomerular basement membranes and diffuse swelling and proliferation of overlying visceral epithelial cells (Jones methenamine silver). (b) In this glomerulus with collapsing FSGS, some of the podocytes are detached from the glomerular basement membrane and lie free within the urinary space (periodic acid Schiff). Magnifications: ×100 in (a) and ×400 in (b).

A 39-year-old male patient had fever, lack of appetite, weakness, waist stiffness for two hours in the morning, recurring oral aphthae, genital ulcers, recurring arthritis particularly at the right knee and bilateral ankles, blurred vision and redness of the right eye and diffuse papulopustular lesions, and erythema nodosum spread over the body (ocular examination revealed uveitis and bilateral papilledema) and was diagnosed with Behçet's disease and neuro-Behçet's disease. He was started on methylprednisolone, colchicine, azathioprine, and cyclosporin A. Because the patient experienced no improvement in ocular complaints despite this treatment for 7 months, azathioprine and cyclosporine A were discontinued and interferon-alpha 6 million units (MU) twice a week was started. He had been treated with interferon-alpha for the past three months without significant side effects. During the follow-up visits, after 12 weeks of initiation of interferon-alpha therapy (cumulative interferon dose was 144 MU) he started complaining of fatigue and scrotal and lower limbs swelling and was admitted for inpatient care. The patient did not report any other chronic illnesses from Behçet's disease or use of other medications, alcohol, or smoking. Family history was irrelevant. With physical examination he had moderate pretibial pitting edema, bilateral scrotal bulge, and minimal ascites. Laboratory values were as follows: creatinine, 274,04 *μ*mol/L; blood urea nitrogen, 28,56 mmol/L; total protein, 39 g/L; albumin, 12 g/L; sedimentation, 74 mm/h; C-reactive protein, 11 mg/L; cholesterol, 8,48 mmol/L; LDL cholesterol, 6,48 mmol/L; triglyceride, 3,25 mmol/L; HDL cholesterol, 0,62 mmol/L; WBC, 6,9 · 10^3^/*μ*L; Hb, 16 g/dL; platelet, 329 · 10^3^/*μ*L. A 24-hour urine analysis demonstrated 7.6 g/day proteinuria. With urinary ultrasonography (USG), there was minimal free fluid between the convolutions at the abdominal lower quadrant; kidney sizes were normal and parenchymal echoes were increased. The pelvicalyceal system was normal. No pathology was noted with renal vessel Doppler USG. Venous blood gas findings were pH 7.306 and HCO3 17.2 mEq/L. Hepatitis markers and HIV serology were negative; autoantibodies (ANA, RF, anti-dsDNA, ENA screen, and ANCA) were also negative. The reported result of the kidney biopsy was consistent with FSGS (Figure 1). Immunofluorescence reveals segmental deposits of Immunoglobulin (Ig) M and C3 in areas of sclerosis and hyalinosis. Electron microscopy reveals severe foot process effacement. There were no proteinuria and kidney failure when he used colchicine, azathioprine, and cyclosporin A. Therefore, interferon-associated FSGS was considered and interferon was discontinued. Treatment with methylprednisolone 1 mg/kg/day and statin and angiotensin-converting enzyme (ACE) inhibitor was started. The patient was monitored during treatment and with the follow-up examination at 3 months, creatinine was decreased to 88,4 *μ*mol/L and 24 h urinary protein was decreased to 0,4 g/day.

## 3. Discussion

FSGS is divided into primary and secondary. While primary or idiopathic FSGS is of unknown cause, secondary FSGS occurs after known cause. The underlying cause is unknown in about 80% of patients [4]. Several pathogenetic mechanisms are effective in primary and secondary types of disease. Changes in the podocyte structure are the basis of ultrastructural signs of the disease. Genetic mutations, circulating glomerular permeability factor, or maladaptive conditions such as hypertension and hyperfiltration in FSGS spectrum of disease cause similar histological and clinical results [5]. Secondary FSGS usually associated with the virus, interferon, lithium, pamidronate and heroin use, mutations which are familially transferred, obesity, hypertension, sickle cell anemia, and so forth is developing in association with adaptive functional structural response due to diseases [4–6]. FSGS clinically manifest with nephrotic syndrome and/or acute renal failure. The disease often has a poor prognosis. It progresses rapidly and may lead to end-stage renal failure [6].

Recombinant interferons are increasingly used in the treatment of malignancy and viral illness [2]. Side effects, both dose-dependent and associated with idiosyncratic toxicity, are seen with interferon. Dose-dependent side effects include flu-like symptoms, leucopenia, anemia, thrombocytopenia, and serum transaminase elevations. These side effects usually resolve with dose reduction [6]. However, hypothyroidism, generalized autoimmune phenomenon, immune-mediated thrombocytopenia, and anemia improve only after treatment discontinuation [7]. Acute renal failure and massive proteinuria have been reported very rarely and can occur any time after the start of interferon therapy and are idiosyncratic toxicities secondary to interferon treatment [8].

The exact mechanism of renal damage associated with interferon is currently unknown. Immune complex glomerulonephritis due to interferon-anti-interferon immune complexes is a theoretical consideration. Among the diverse speculations to explain the etiological role of interferon causing severe proteinuria, it is tempting to consider a sequence where interferon may participate in alteration of protein glomerular permeability either directly or through release of other cytokines by activating T lymphocytes [9]. In an experiment by Bino et al., crescentic glomerulonephritis was shown by injecting interferon to newborn mice. IgG, complement, and, in some places, IgM storage was demonstrated with the biopsy material obtained [10].

Individuals with genetic variants in the Apolipoprotein L1 (APOL1) gene have greatly increased risk of kidney disease. The high-risk genotypes are associated with elevated risk (7–29-fold) of FSGS, hypertension-associated end-stage renal disease, and HIV-associated nephropathy [11, 12]. Only individuals with recent African ancestry carry these risk variants, explaining a large part of the 4-5-fold increased rate of kidney disease in African Americans compared with Caucasians [11, 12]. Nichols et al. reported a cohort of patients who developed collapsing focal segmental glomerulosclerosis while receiving therapeutic interferon, all of whom carried the* APOL1* high-risk genotype. This finding raised the possibility that interferons and the molecular pattern recognition receptors that stimulate interferon production may contribute to* APOL1*-associated kidney disease. In cell culture, interferons and toll-like receptor agonists increased* APOL1* expression up to 200-fold. PolyI:C, a double-stranded RNA TLR3 agonist, increased* APOL1* expression by upregulating interferons directly or through an interferon-independent pathway with another pathway like NF-*κ*B and jak kinases [11]. Although our patient is not African, FSGS developed after using interferon treatment.

There is no proven treatment for renal toxicity associated with interferon. The common practice is to cease interferon and administer steroids. While renal functions recover following treatment discontinuation in some cases, renal functions remain unchanged and even progress to end-stage renal failure in others. The factors involved in this process are yet unknown. Case-based evaluation indicates a relationship between renal functions and degree of collapse and sclerosis of the glomeruli and the presence of comorbidities including diabetes and hypertension [13].

FSGS treatment is important, firstly excluding secondary causes that can lead to disease and determining the bad prognostic factors. While proteinuria amount is 0.5–2 g/day with conventional treatments, for nephrotic-range proteinuria, renal function impairment, and presence tubulointerstitial damage in biopsy aggressive treatment is recommended. Treatment options include blood pressure control, use the ACE inhibitors, statins, corticosteroids, cytotoxic drugs (cyclophosphamide and chlorambucil), calcineurin inhibitors (cyclosporine and FK 506), mycophenolate mofetil, vitamin E, perfloxacin, plasmapheresis, and immunoadsorption [5, 14].

In conclusion the fact that patients with FSGS associated with interferon have different disease processes and respond differently to discontinuing interferon and commencing steroid treatment indicates that further studies are needed for a better understanding of the underlying pathogenesis and complication management.
